# Plasma markers of neurodegeneration, latent cognitive abilities and physical activity in healthy aging

**DOI:** 10.1038/s41598-024-72806-0

**Published:** 2024-09-17

**Authors:** Jonna Nilsson, Yiwen Jiang, Malin Johannesson, Marcus Moberg, Rui Wang, Susanne Fabre, Martin Lövdén, Örjan Ekblom, Maria Ekblom

**Affiliations:** 1 Swedish School of Sport and Health Sciences, Stockholm, Sweden; 2 Aging Research Center, Karolinska Institutet and Stockholm University, Stockholm, Sweden; 3 BioArctic AB, Stockholm, Sweden; 4 Department of Neurobiology, Care Sciences and Society, Karolinska Institute, Stockholm, Sweden; 5 Wisconsin Alzheimer’s Disease Research Center, University of Wisconsin School of Medicine and Public Health, Madison, USA; 6 Department of Psychology, University of Gothenburg, Gothenburg, Sweden

**Keywords:** Cognitive aging, Physical activity, Neurofilament light, NfL, Amyloid beta, Phosphorylated-tau, Cognitive ageing, Biomarkers

## Abstract

Blood-based biomarkers of neurodegeneration demonstrate great promise for the diagnosis and prognosis of Alzheimer’s disease. Ultra-sensitive plasma assays now allow for quantification of the lower concentrations in cognitively unimpaired older adults, making it possible to investigate whether these markers can provide insight also into the early neurodegenerative processes that affect cognitive function and whether the markers are influenced by modifiable risk factors. Adopting an exploratory approach in 93 healthy older adults (65–75 years), we used structural equation modelling to investigate cross-sectional associations between multiple latent cognitive abilities (working memory, episodic memory, spatial and verbal reasoning) and plasma amyloid beta (Aβ42/Aβ40 ratio), phosphorylated-tau 181 (ptau-181), glial fibrillary acidic protein (GFAP), and neurofilament light (NfL), as well as the influence of device-measured habitual physical activity on these associations. The results showed that NfL was negatively associated with working memory, and that NfL interacted with moderate-to-vigorous physical activity in its association with episodic memory. The study has thereby demonstrated the potential of neurodegenerative plasma markers for improving understanding of normative cognitive aging and encourages future research to test the hypothesis that high levels of NfL, indicative of white matter pathology, limit the beneficial effect of physical activity on episodic memory in healthy aging.

Blood-based biomarkers of neurodegeneration have emerged as a groundbreaking tool for the diagnosis and prognosis of Alzheimer’s disease (AD), as well as for improving the design of intervention trials^1^. Relative to cerebrospinal fluid (CSF) biomarkers and molecular neuroimaging, blood plasma can be obtained much less invasively and at a lower cost, enabling measurement of plasma biomarkers in a broader range of settings. Plasma amyloid beta (Aβ42/Aβ40 ratio), phosphorylated-tau 181 (ptau-181), glial fibrillary acidic protein (GFAP), and neurofilament light (NfL) are examples of putative blood-based biomarkers for progression of disease mechanisms relevant to AD^2,3^. As such, these markers are not only altered across the AD continuum but also prospectively predict cognitive decline, PET- Aβ load, and conversion to AD^4–6^.

The prognostic use of blood-based biomarkers for AD is possible due to neurodegenerative processes that start long before cognitive symptoms emerge^7^. Specifically, AD typically starts with a buildup of amyloid beta plaques (Aβ) followed by an accumulation of aggregated phosphorylated tau (p-tau), as early as in midlife and decades before diagnosis^7^. Similarly, astrocytic damage or activation around the Aβ plaques, as indicated by GFAP, also emerge at the pre-symptomatic stage of AD^8^. NfL is released upon neuro-axonal injury and is elevated in several neurological diseases, reflecting its role as a more non-specific marker of neurodegeneration^9^. Nevertheless, NfL also predicts cognitive decline and progression to AD in cognitively unimpaired older adults^5^, possibly independently of Aβ pathology^10^. Taken together, Aβ, p-tau, GFAP and NfL, can be detected also in cognitively intact older adults, albeit at lower average concentrations^4^. Recently, ultra-sensitive plasma assays, such as the single molecule enzyme-linked immunosorbent assay (Simoa) and the Mesoscale Discovery (MSD), have come to allow for much lower protein concentrations to be measured in blood plasma^11^. Given such technical advancement, blood-based biomarkers therefore have the potential of providing insights also into the very early and more subtle neurodegenerative processes that may influence cognitive function in healthy aging.

The preclinical stage of AD has been identified as an opportunity to intervene, before substantial neurodegeneration and cognitive impairments are realized^12^. Immense pharmacological research efforts to prevent and modify the AD disease process are underway^13^, with anti-amyloid antibody therapies in early AD demonstrating particular promise, with Lecanemab being approved in the U.S., Japan, and China^14,15^. A parallel effort has been to identify potentially modifiable risk factors that can slow neurodegeneration and prevent AD, with physical activity gaining particular attention^16–18^. Prospective studies have indeed demonstrated that higher levels of physical activity are robustly associated with a decreased risk of age-related cognitive decline and dementia^19–21^. Unfortunately, however, cognitive effects of physical exercise interventions in randomized-controlled trials (RCT) have been mixed for AD^22–24^ and an umbrella review of meta-analyses recently concluded lacking evidence for a benefit in older age^25^. One possible explanation for this discrepancy is that the protective mechanisms of physical activity might require longer time periods to emerge, where prospective studies may reflect physical activity habits over longer time periods than the 20-week intervention duration that is typical for RCTs in older adults^25^.

One of the many proposed hypotheses for the protective role of physical activity is that it alters the neurodegenerative processes that underlie age-related cognitive decline and AD, such as the accumulation of Aβ and p-tau^26,27^. Whilst animal studies have provided experimental support of such a mechanism^28,29^, rigorous RCTs in humans with long intervention periods are largely lacking^26^. Evidence from observational studies has also not been consistent in demonstrating associations between physical activity and neurodegenerative markers, such as Aβ, p-tau and NfL in cognitively intact older adults^30–33^. Furthermore, only a limited body of work has directly probed the interactive associations of physical activity and neurodegenerative markers with cognition. Rabin, et al.^34^ reported an interaction between pedometer-measured physical activity with PET-Aβ burden in healthy older adults, such that greater physical activity was associated with slower Aβ-related cognitive decline. Similarly, Desai, et al.^35^ found evidence for an interaction between blood-based total tau and self-reported physical activity in a population-based cohort of older adults, such that higher levels of physical activity and low tau were associated with the greatest reduction in subsequent cognitive decline. In another study using the same cohort, Desai, et al.^36^ found that at low NfL concentrations, moderate levels of physical activity exhibited a stronger association with slower global cognitive decline, whilst at high NfL concentrations, high levels of physical activity were more tightly coupled to slower cognitive decline. Similarly, Raffin, et al.^32^ found that high plasma NfL concentrations were associated with a dampening of the favorable association between self-reported moderate-to-vigorous physical activity and cognition in community-dwelling older adults, with no associations for plasma Aβ42/Aβ40 ratio. Taken together, the interactive associations of physical activity and neurodegenerative markers with cognition in old age is not yet clear.

From the summary above, it is also worth noting that previous research that has targeted the interaction between neurodegenerative biomarkers and physical activity with cognition in normal aging have suffered some limitations. Measures of physical activity have often relied on self-report, which is subject to recall and social desirability biases, and studies have typically been restricted to only one or two neurodegenerative markers, hindering a broader evaluation of different markers’ relative importance for the association between physical activity and cognition^32,35–37^. Furthermore, previous studies have tended to use a global composite score for cognition and based it on a smaller set of tests, in which brevity and sensitivity to clinically relevant cognitive decline is prioritized over a more exhaustive assessment distinguishing different cognitive abilities^32,34–37^.

In the present study, we capitalize on the rich baseline data of an already completed RCT conducted in healthy older adults (Nilsson et al., 2020), to explore the cross-sectional associations between blood-based markers of neurodegeneration, physical activity as a modifiable lifestyle factor, and cognition. Building on our previous work^38^, we used structural equation modeling to model multiple theoretically error-free latent cognitive abilities from a large number of tests, designed to capture variation of cognitive ability in the normal range. Accelerometers were used to objectively measure habitual physical activity patterns at different intensities, and four different neurodegenerative blood-based biomarkers were quantified using ultra-sensitive plasma assays. The first aim was to explore the relative importance of Aβ42/Aβ40 ratio, ptau-181, GFAP and NfL for episodic memory, working memory, spatial reasoning and verbal reasoning in healthy older age. The second aim was to probe whether accelerometer-measured physical activity interacts with the neurodegenerative markers in their association with latent cognitive abilities. Given its exploratory nature, the overall purpose of the study was to contribute to the generation of *new* hypotheses concerning the role of neurodegenerative markers and its interaction with physical activity for cognition in healthy aging.

## Methodology

### Participants

97 participants between 65 and 75 years without serious physiological or psychological illness completed the original study (Nilsson et al., 2020). Participants were screened for dementia and mild cognitive impairment using the Mini Mental State Exam^39^, including only those with a score of 26 or higher. The original study was approved by the ethical review board in Stockholm (Regionala Etikprövningsnämnden, Stockholm, case number 2017/1115-31/4) and conducted in accordance with the Declaration of Helsinki. The extended blood analysis was later approved by the same authority (case number 2018/329-32). Only the 93 participants who provided informed consent to the extended blood analysis were included in the present study.

## Design and procedure

### Blood sampling and plasma analysis

Only blood samples collected at rest at the baseline assessment were used in the present study. A peripheral intravenous catheter was inserted into the antecubital vein upon arrival and after 15 min of seated rest, the at-rest sample was drawn. The session was scheduled for the first half of the day (08:00–12:30), with the at-rest sample being collected first. For details on the complete blood sampling protocol, please refer to our previous work^40,41^.

For the at-rest sample, 5 mL was collected into heparinized containers for plasma analysis. The blood was spun at 4 °C for 10 min at 3000 g to separate the plasma. All samples were then placed in a new container and frozen at − 80 °C. The samples have subsequently been subject to three freeze–thaw cycles for previous purposes. Plasma samples were again thawed at room temperature and centrifuged at 10,000 g for 5 min at 4 °C to remove debris. Subsequently, plasma GFAP, NfL and ptau-181 concentrations were analyzed in duplicates using the Simoa Neurology 2-plex B kit (Quanterix, USA) and the Simoa ptau-181 kit (Quanterix, USA) respectively in accordance with manufacturer guidelines. Plasma Aβ38, 40, 42 were measured using the V-PLEX Aβ Peptide Panel 1 (6E10) kit (MSD, USA) in duplicates according to manufacturer guidelines. To assess the effect of freeze–thaw cycles on the concentrations of the above proteins, a pre-experiment was performed. In brief, fresh plasma samples from eight healthy individuals were processed for one, two, or three freeze–thaw cycles respectively and the corresponding concentrations of Aβ38, 40, 42, GFAP, NfL and ptau-181 for each cycle were measured using the same methods as in the main experiment (SM-1). All proteins appeared stable across freeze–thaw cycles, which is in line with previous demonstrations of stability of these particular plasma markers over three freeze–thaw cycles^42^.

Mean concentrations were used for duplicate measurements, and samples with a coefficient of variation above 20% were excluded, in line with manufacturer guidelines. As a result, data for 12 samples were excluded due to excessive variation (1 Aβ40, 3 ptau-181, 1 NfL, 7 GFAP). In addition, in four samples, only a single measurement was generated, which were also not included in the analyses (4 NfL, 4 GFAP). Lower limit of quantification was 29.9 pg/mL for Aβ40, 3.3 pg/mL for Aβ42, 8.9 pg/mL for ptau-181, 16.6 pg/mL for GFAP, and 1.6 pg/mL for NfL.

### Cognitive measures

In the original investigation, the cognitive assessment included 18 tests, which were developed to target a wide range of pre-specified cognitive domains. As well as allowing for a multifaceted approach to cognition, the inclusion of more than one test per domain allowed for cognitive ability to be modelled in latent space (see Statistical Analysis). In the present analyses, we build on the same cognitive measurement model that was carried forward in our previous work^38^. This model incorporated four of the six cognitive domains intended for the original study, with the domains of processing speed (2 tests) and working memory switching (2 tests) being abandoned due to Heywood cases. Thus, the cognitive model applied here incorporated 14 computer-based cognitive tests targeting four different domains: working memory updating (2 n-back tests, 2 running span tests, 2 updating tests), episodic memory (1 verbal recall test, 1 spatial recall test), spatial reasoning (3 matrix reasoning tests) and verbal reasoning (1 analogies test, 1 syllogisms test, 1 verbal comprehension test). Detailed descriptions of all the cognitive tests can be found in the supplementary materials of the original publication^40^.

### Accelerometry

Physical activity was measured using accelerometers worn in a waist belt over the hip for 7 days before the first study visit (Actigraph GT3X + , Actigraph LCC; Pensacola, FL, USA). Accelerometer data were recorded as raw data (sample rate set to 30 Hz) from all three axes, which were combined into a resulting vector, and extracted as 60 s epochs. A low-frequency filter was used, which increases the sensitivity at lower intensity activities and allows for better detection of slower steps in the elderly population^43^. Using standard definitions, moderate-to-vigorous intensity physical activity (MVPA) was defined as ≥ 2690 counts per minute, light-intensity physical activity (LIPA) as 200 to 2689 counts per minute, and sedentary behavior (SED) as 0–199 counts per minute^44,45^. A minimum of 600 min of valid daily wear time for at least 4 days was required to be included in the analyses^46^, and wear-time was calculated using the Choi algoritm^47^. To account for variability in wear-time, total time spent in MVPA (min) was divided by total wear-time (min) for each participant resulting in a proportion of time spent in MVPA for each day. Although such a correction can influence results, a high correlation between adjusted and unadjusted MVPA in the present study makes such a scenario unlikely, *r*(91) = 0.99, *p* < 0.0001. All analyses were therefore conducted using MVPA adjusted for wear-time.

### Fitness

Maximum rate of oxygen consumption (VO_2_max) was measured using a maximal treadmill ergometer and was used here for characterization of the study population. The maximal test has been described in detail elsewhere^38,41^, but briefly it started with an initial treadmill incline of 1 degree at a comfortable speed and continued with increases of incline and/or speed every minute until volitional exhaustion. VO_2_ max was measured using a computerized metabolic system (Jaeger Oxycon Pro, Hoechberg, Germany), expressed in relative (mL/kg/min) units.

## Statistical analysis

### Plasma neurodegenerative markers

Aβ42/40 ratios were calculated by dividing Aβ42 concentrations with Aβ40 concentrations. Descriptive statistics were generated for all neurodegenerative markers, including Aβ42, Aβ40 and the Aβ42/40 ratio. Subsequent analyses proceeded with Aβ42/40 ratio, ptau-181, NfL and GFAP. Considering the exploratory nature of the investigation, a cautious approach for detecting and handling outliers was warranted. To this end, the outlier labelling rule was applied with a conservative constant of 3 times the interquartile range to identify only the most extreme outliers, and analyses were performed and reported with and without outliers when relevant.

Zero-order Pearson’s correlations were calculated for descriptive purposes, for associations among the four markers, as well as between markers and physical activity at the different intensity ranges. Pearson’s correlation analyses were also conducted for the markers and selected continuous variables (age, MMSE, BMI, MVPA, fitness). To also describe the influence of selected categorical demographic variables, Welsh’s t-tests (sex) and one-way ANOVAs (educational level) were additionally conducted for all four markers.

### Structural equation modelling

Structural equation modelling was performed using the ‘sem’ function in the lavaan package (version 0.6.16; Rosseel, 2012), run in the R programming environment (version 4.3.2; R Core Team, 2014). All cognitive variables were approximately normally distributed and were standardized and converted into T scores, with a mean of 50 and a standard deviation of 10, before being used for modelling. Model fit was evaluated using the Comparative Fit Index (CFI) and the Root Mean Square Error of Approximation (RMSEA), where models with RMSEA of less than 0.06 and CFI of 0.95 or higher were judged as having sufficiently satisfactory fit for interpretation (Hu & Bentler, 2009). Full-information maximum likelihood estimation was used to deal with missing data. In all models, the variances of the latent factors were fixed to unity and all factor loadings were freely estimated. Statistical significance of the parameters of interest was assessed using χ2 -difference tests, comparing model fit when the path of interest was constrained to zero with the same association when estimated freely. The significance threshold for the χ2 -difference tests was set to *p* = 0.05. Importantly, significance was interpreted only as an indication that the information may be useful for generation of *new* hypotheses, and not as confirmatory support of any a priori hypotheses. For significant parameters, standardized coefficients were reported together with their respective 95% confidence intervals (CI).

**Cognitive model:** All presented models built on the same cognitive measurement model that was pursued in our previous work^38^. As such, four cognitive ability factors were modelled (episodic memory, working memory, spatial reasoning, verbal reasoning), allowing the cognitive ability factors to correlate with each other. Modelling abilities in latent space like this allows for attenuation of reliability problems of raw scores and allows for task-specific variance to be reduced in favor of task-general (ability) variance. Considering that the study population and thus the underlying data in the present study differed slightly from that in our previous work, the cognitive measurement model was fitted and reported on again.

**Plasma neurodegenerative marker prediction model:** To investigate the relative strength of the associations between plasma neurodegenerative markers and cognition, all four markers were entered as predictors of the latent cognitive abilities in the cognitive model. By allowing the markers to correlate, each path was a partial regression with respect to the other markers. This means that the estimated paths reflect the unique contribution of each individual marker to cognition, above and beyond associations with the other markers. For completeness, the markers were also entered as predictors one by one, probing also non-unique relations between the neurodegenerative markers and cognition. To help with model conversion, all neurodegenerative marker variables were scaled to have a mean of zero and a standard deviation of 1 in order to handle otherwise large variances relative to the other variables in this particular model.

**Physical activity interaction model:** In a final step, a regression interaction approach was adopted to explore a potential interaction between MVPA and NfL in their relationship with cognition. The motivation for this model was twofold: first, the significant zero-order correlation between NfL and MVPA, and second, that NfL was the only neurodegenerative marker that was associated with cognition in the previous prediction model. For the interaction model, an interaction term was calculated between NfL and MVPA, mean-centering the variables before multiplication to avoid the linear dependency in the covariance matrix that result from cross-multiplying raw scores (Kline et al., 2000). The resulting interaction term was then mean-centered again to allow for visualization of the moderating effect (see below). The double-centered interaction term was then entered as a predictor in the cognitive model, along with NfL and MVPA (no scaling). The three predictors were allowed to correlate, meaning that associations between the interaction term and cognition represented the contribution of the interaction above and beyond the main effects of NfL and MVPA. The same modelling approach was subsequently repeated for SED and LIPA, to additionally probe the moderating role of physical activity at the lower end of the intensity spectrum.

**Visualization of the interaction:** The combined effects of the predictors in a regression interaction model with latent elements can be difficult to ascertain by the path coefficients alone. To gain a better understanding of the nature of the moderation, we therefore created a visualization using subsets of data laid out across multiple plots in R (version 4.3.2), following the procedure proposed and described by Hallgren, et al.^48^. Variable estimates were first extracted from a confirmatory factor analysis, based on a model identical to the physical activity moderator model, except that it did not include the interaction term and no structural regressions. Using the extracted variable estimates (*M* = 0, *SD* = 1), simple slopes were then computed using the ‘lm’ function, with the cognitive ability of interest as dependent variable and the model predictors as independent variables (e.g. NfL, MVPA, NfL x MVPA interaction). For each quartile of NfL, the ‘predict’ function was subsequently used to estimate the predicted value for the latent cognitive ability of interest for different values of MVPA, visualizing the influence of NfL as the moderator. The visualization was also performed using MVPA as moderator, plotting the predicted value for the latent cognitive ability for different values of NfL, for each quartile of MVPA. It is important to note that the two visualizations are mathematically equivalent but can nevertheless generate different substantive interpretations.

**Sensitivity analysis:** Considering the explorative aim of the present study, relevant confounders had not been identified a priori. To minimize the risk of introducing more bias than is removed by statistical adjustment, a conservative approach to model adjustment was adopted, including only age, sex and education as covariates for the sensitivity analysis. To this end, the co-variates were regressed on the cognitive ability factors, allowing them correlate amongst themselves and with the other predictors in the model. Sensitivity analyses were performed after excluding detected extreme outliers in the neurodegenerative marker variables. Considering the detected association between NfL and BMI, an extended sensitivity analysis was also conducted, in which BMI was added to the original set of covariates.

## Results

Results include (1) descriptive results in regard to the study population and the plasma neurodegenerative markers, and (2) structural equation models targeting associations between latent cognitive abilities and the plasma neurodegenerative markers, the role of physical activity for these associations, as well as sensitivity analyses.

## Descriptive results

### Participant characteristics

All study participants were cognitively unimpaired when entering the study (MMSE ≥ 26). Table 1 contains additional study sample characteristics, including information regarding demography, cognitive status, physical activity, and physiology.

**Table 1:** Descriptive statistics for the study population.

		n	Mean	sd
Demographics	Age (year)	93	70.42	2.94
	Sex (female/male)	47/46	–	–
	Education (level 1/2/3)	8/16/66	–	–
Cognitive status	MMSE (score)	93	29.39	0.92
Physical activity*	Moderate-to-vigorous (proportion)	91	0.06	0.02
	Light-intensity (proportion)	88	0.38	0.08
	Sedentary (proportion)	91	0.56	0.09
Physiology	BMI (kg/m^2^)	88	25.71	3.73
	VO_2_ max (mL/kg/min)	90	31.52	5.38

Education level: 1 = elementary school, 2 = high school, 3 = university. MMSE: Mini Mental State Exam.

*Average proportion daily accelerometer-measured time spent in physical activity during the 7-day assessment (min), expressed in proportion of wear time (min).

### Plasma neurodegenerative markers

**Fig. 1:**
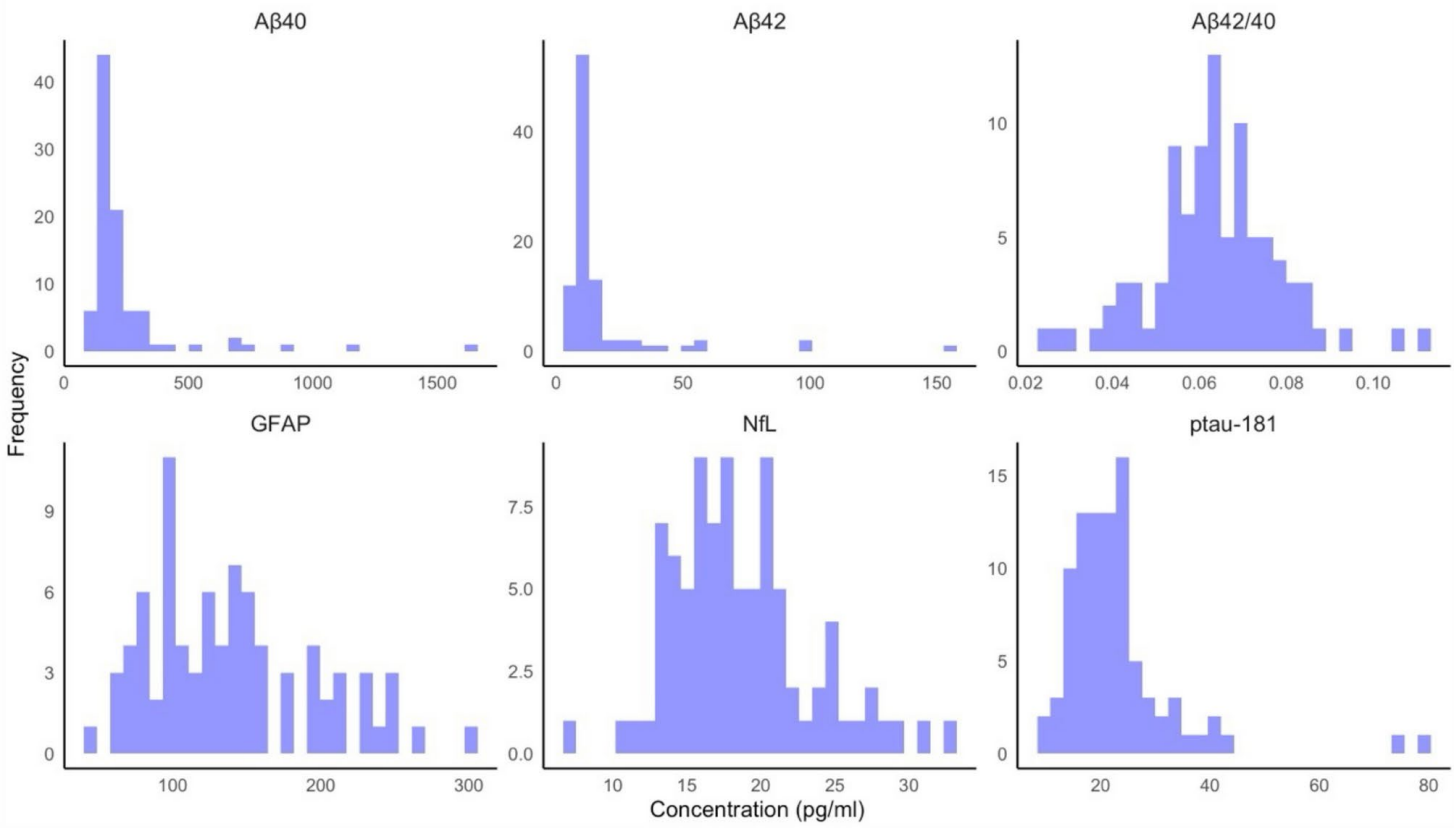
Histograms of the plasma neurodegenerative marker distributions.

Descriptive statistics and distributions for plasma concentrations of the quantified neurodegenerative markers can be found in Table 2 and Fig. 1, respectively. Adopting the outlier labelling rule (*IQR* = 3.0) to detect extreme values, seven outliers were detected for Aβ40. No other extreme outliers were detected.

**Table 2:** Plasma concentrations of the neurodegenerative markers in pg/mL.

Neurodegenerative marker	n	Mean	SD
Aβ40	92	245.48	219.81
Aβ42	93	16.95	21.35
Aβ42/40 ratio	92	0.06	0.02
ptau-181	90	22.91	10.61
NfL	88	18.60	4.71
GFAP	82	139.22	56.31

Correlation analyses for the neurodegenerative markers Aβ42/40, GFAP, NfL and ptau-181 revealed one statistically significant correlation between Aβ42/40 and NfL, *r*(85) = 0.22, *p* = 0.041 (95% CI [0.01, 0.41]). However, this correlation was not significant when excluding the seven subjects with extreme values for Aβ40, *r*(79) = 0.10, *p* = 0.35 (95% CI [− 0.12, 0.32]). Regarding correlations between the markers and physical activity (MVPA, LIPA, SED), one significant correlation was detected between higher NfL concentrations and higher levels MVPA, *r*(84) = 0.21, *p* = 0.048, (95% CI [0.002, 0.41]). Of note, this association was no longer significant when adjusting for age, sex and education (*p* = 0.14). For complete correlation matrix, see SM-2.

Regarding continuous demographic variables (age, BMI, MMSE), correlation analyses revealed three significant correlations. NfL concentrations were negatively correlated with BMI, *r*(81) =  − 0.23, *p* = 0.040, (95% CI [− 0.42, − 0.01]). Furthermore, Aβ42/40 concentrations were correlated with MMSE, *r*(90) =  − 0.23, *p* = 0.031, (95% CI [− 0.42, − 0.02]), but this associations was no longer significant when excluding the subjects with extreme values for Aβ40, *r*(83) =  − 0.14, *p* = 0.22 (95% CI [− 0.34, 0.08]). For complete correlation matrix, see SM-2. Regarding categorical demographic variables (sex, education), only GFAP differed by sex (mean difference = 38.4), being greater for females (*M* = 157.46) than males (*M* = 119.1), *t*(79.46) = 3.29, *p* = 0.002 (95% CI [15, 62]).

## Structural equation modelling

### Cognitive model

This cognitive model demonstrated satisfactory fit, *χ2* (71, *N* = 93) = 93.68, *CFI* = 0.96, *RMSEA* = 0.059, with moderate-high loadings of observed test variables on the latent cognitive ability factors (*β* = 0.48–0.88) and moderate-high covariances among the four cognitive ability factors *(β* = 0.39–0.82). This model formed the basis for all subsequent models.

### Plasma neurodegenerative marker prediction model

**Fig. 2:**
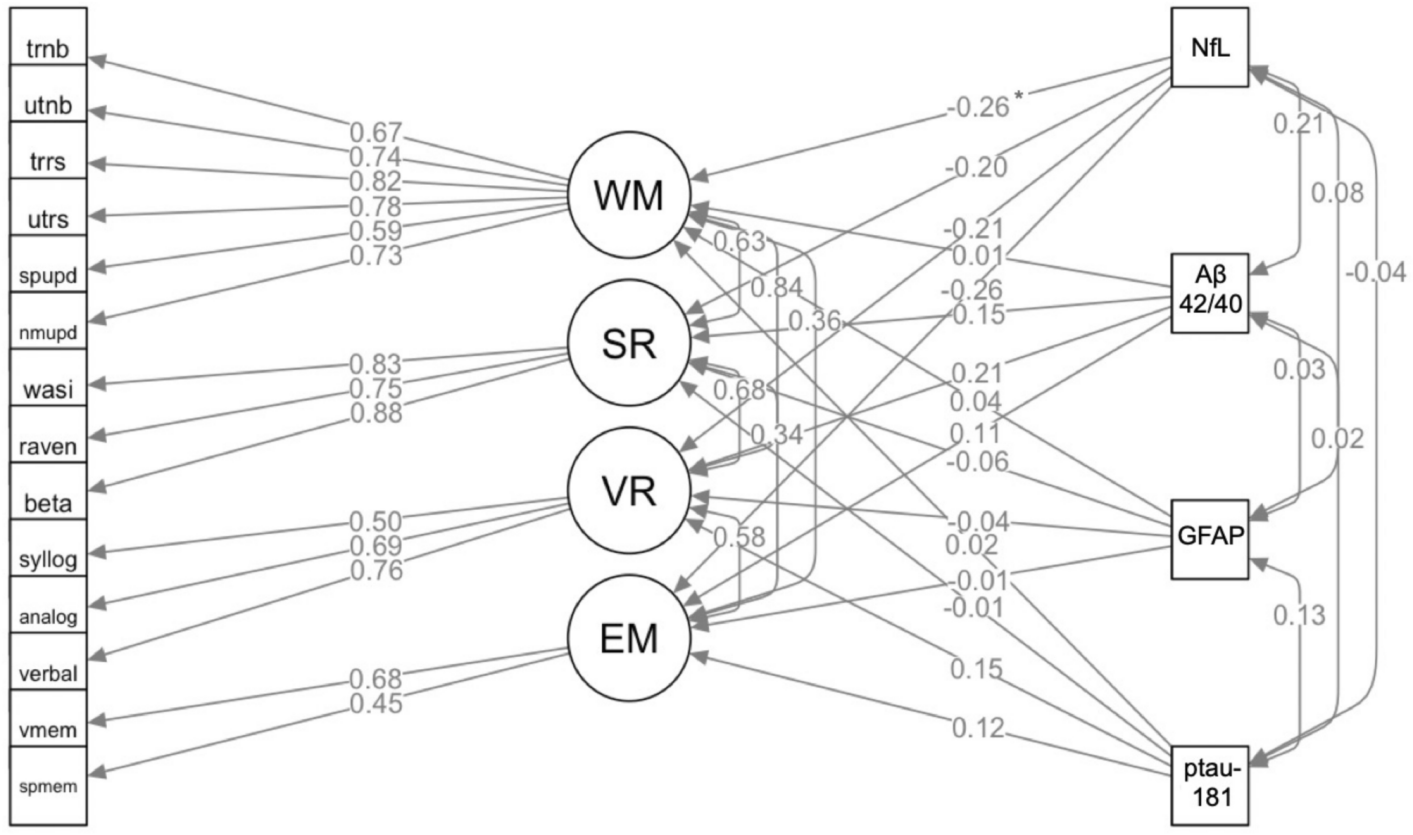
Plasma Neurodegenerative Marker Prediction Model. Latent variables are presented in circles and observed variables in rectangles. Standardized regression weights (βs) are represented by single-headed arrows and co-variances by double-headed arrows. The variances of all latent variables were fixed to unity and all factor loadings, intercepts, variances, and covariances were estimated. MVPA = proportion time in moderate-to-vigorous physical activity relative to wear-time, WM = working memory updating, SR = spatial reasoning, VR = verbal reasoning, EM = episodic memory. For abbreviations of individual observed variables (tests) and their detailed descriptions, see Nilsson, et al.^38^. Paths marked with * were significant (*p* < .05).

The neurodegenerative marker prediction model demonstrated satisfactory fit, *χ2* (153, *N* = 93) = 115.95, *CFI* = 0.99, *RMSEA* = 0.021 (see Fig. 2 for all standardized parameter estimates). The path between NfL and working memory was negative and significant, *χ2* (1, *N* = 93) = 4.71,* p* = 0.030 (*β* =  − 0.26, 95% CI [− 0.48, − 0.04]), reflecting an association between higher NfL concentrations and worse working memory ability. No other paths between markers and cognitive abilities were significant, but it can be noted that path estimates were negative also between NfL and episodic memory, *χ2* (1, *N* = 93) = 2.89, *p* = 0.09 (*β* =  − 0.26, 95% CI [− 0.55, 0.02]), spatial reasoning, *χ2* (1, *N* = 93) = 2.92, *p* = 0.09 (*β* =  − 0.20, 95% CI [− 0.42, 0.02]), and verbal reasoning, *χ2* (1, *N* = 93) = 2.85, *p* = 0.09 *(β* =  − 0.21, 95% CI [− 0.45, 0.03]). Of note, when excluding Aβ42/40 ratio values for subjects with extreme values for Aβ40, model fit remained satisfactory, *χ2* (153, *N* = 93) = 124.97, *CFI* = 0.97, *RMSEA* = 0.037, and the negative path between NfL and working memory was still significant, *χ2* (1, *N* = 93) = 5.04, *p* = 0.025 (*β* =  − 0.26, 95% CI [− 0.48, − 0.04]). For completeness, each neurodegenerative marker was also added as the single predictor in the cognitive model. All models had satisfactory fit (*RMSEA* < 0.06, *CFI* > 0.95) and showed the same pattern of results, namely the one significant path between NfL and working memory, χ2 (1, N = 93) = 4.86, *p* = 0.028 (*β* =  − 0.25, 95% CI [− 0.46, − 0.04].)

Taken together, based on the available data and conducted analyses, NfL emerged as the only neurodegenerative marker with a reliable association with cognition, in addition to being the only marker demonstrating a zero-order correlation with physical activity, specifically MVPA. This motivated the final model, which tested whether NfL and MVPA interact in their association with cognition.

### Physical activity interaction model

**Fig. 3:**
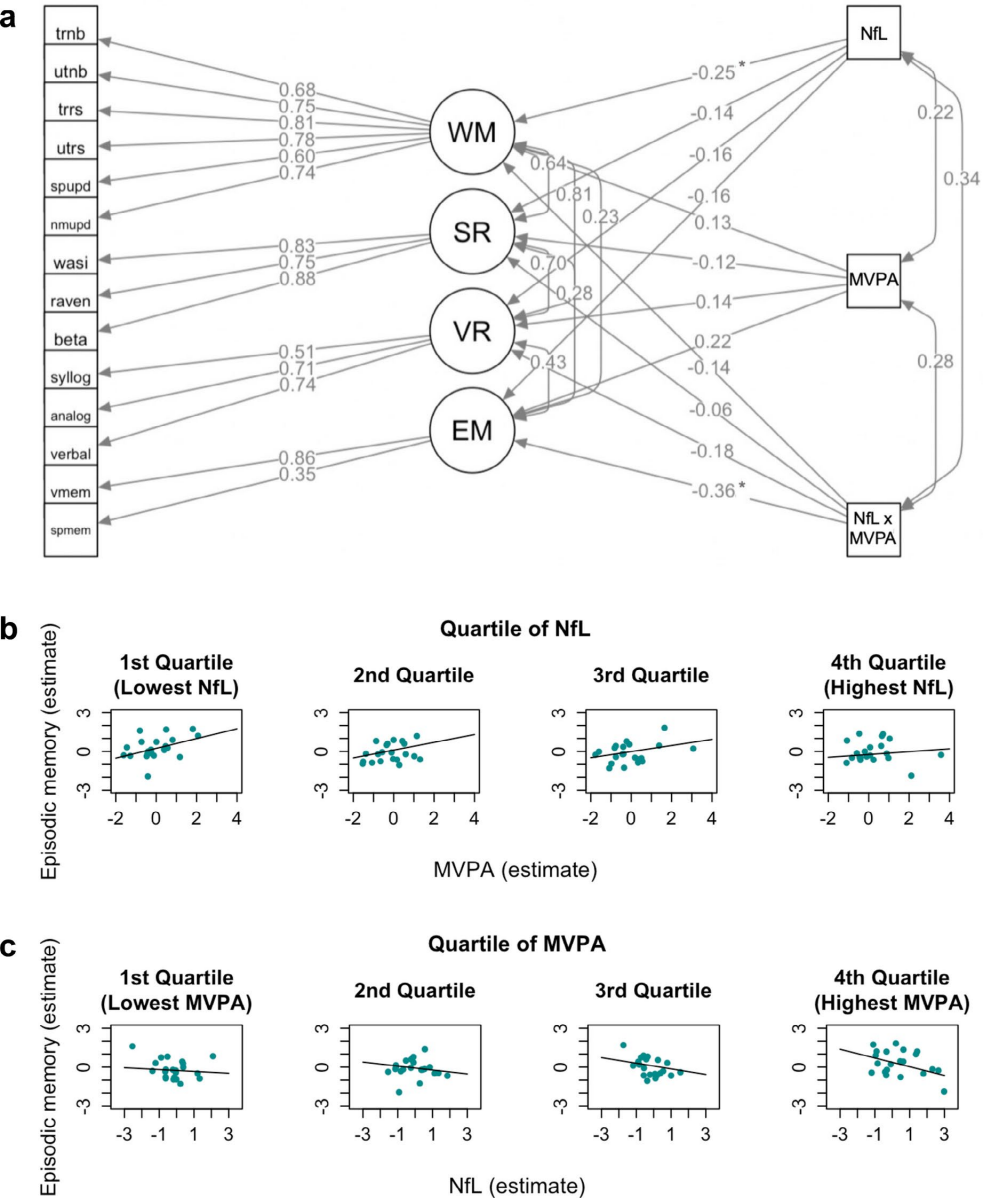
Physical Activity Intraction Model (**A**) and visualization of the significant interaction between NfL and MVPA on EM (**B**). In 3a, latent variables are presented in circles, observed variables in rectangles, and standardized regression weights (βs) are represented by single-headed arrows and co-variances by double-headed arrows. MVPA = proportion time in moderate-to-vigorous physical activity relative to wear-time, NfL = plasma concentration neurofilament light, NfL x MVPA = interaction term for NfL and MVPA, WM = working memory updating, SR = spatial reasoning, VR = verbal reasoning, EM = episodic memory. For abbreviations of individual observed variables (tests) and their detailed descriptions, see Nilsson, et al.^38^. Paths marked with * were significant (*p* < .05). In Fig. 3b, estimates for the latent episodic memory are plotted against estimates for MVPA for different quartiles of NfL, visualizing the significant path from NfL x MVPA to EM in Fig. 3a. In Fig. 3c, the same path is visualized but with estimates for the latent episodic memory plotted against estimates for NfL for different quartiles of MVPA. Note that the points in Fig. 3b,c represents model-predicted values and not actual data points.

When NfL and MVPA were entered together with their interaction term to the cognitive model, model fit was again satisfactory, χ2 (101, N = 93) = 120.10, CFI = 0.97, RMSEA = 0.05 (see Fig. 3a for all standardized parameter estimates). The path from the interaction term to episodic memory was significant, *χ2* (1, *N* = 93) = 6.40, *p* = 0.011, reflecting an interaction between NfL and MVPA in their association with episodic memory (*β* =  − 0.36, 95% CI [− 0.48, − 0.11]). When visualized with NfL as the moderator, this interaction effect appeared to be reflective of a stronger positive association between episodic memory and MVPA at lower levels of NfL (Fig. 3b). When instead visualized with MVPA as the moderator, the interaction effect appeared to reflect a strengthening of the negative association between NfL and episodic memory at higher levels of MVPA (Fig. 3c). As in the previous model (Fig. 2), the path between NfL and working memory was again significant, *χ2* (1, *N* = 93) = 4.26, *p* = 0.039, with a negative path estimate (*β* =  − 0.25, 95% CI [− 0.48, − 0.02]). Of note, as in the correlational analysis, the positive covariance between MVPA and NfL was also significant, *χ2* (1, *N* = 93) = 4.25, *p* = 0.039 (*β* = 0.22, 95% CI [0.02, 0.42]). No other regression paths were significant, but the positive path estimate between MVPA and episodic memory is worth noting, *χ2* (1, *N* = 93) = 3.30, *p* = 0.069 (*β* = 0.22, 95% CI [− 0.05, 0.48]). The same model was subsequently fitted for LIPA and SED, but these models did not have satisfactory fit and were therefore not interpreted further (*RMSEA* > 0.06, *CFI* < 0.95).

### Sensitivity analyses

When the plasma neurodegenerative marker prediction model was adjusted for age, sex and education, model fit was still satisfactory, χ2 (141, N = 93) = 168.99, CFI = 0.95, RMSEA = 0.046. The previously detected negative path between NfL and working memory was no longer significant, χ2 (1, N = 93) = 4.42, *p* = 0.065, but with a similarly sized standardized estimate (β =  − 0.22, 95% CI [− 0.48, 0.01]). The other regression paths were non-significant.

When the physical activity interaction model was adjusted for age, sex and education, model fit was satisfactory, χ2 (134, N = 93) = 164.87, CFI = 0.95, RMSEA = 0.050. The previously detected negative path between the interaction term between NfL and MVPA on episodic memory was still significant, χ2 (1, N = 93) = 10.04, *p* = 0.002, with a similarly sized standardized estimate, β =  − 0.38, 95% CI [− 0.62, − 0.13]. Of note, the covariance between MVPA and NfL was also significant, χ2 (1, N = 93) = 4.07, *p* = 0.044, with a positive path estimate (β = 0.21, 95% CI [0.01, 0.41]).

Considering the inverse relationship between NfL and BMI, additional sensitivity analyses were conducted with BMI added to the covariate set. The outcome of the analyses did not change for any of the two models (SM-3).

## Discussion

The present study adopted an exploratory approach to investigate cross-sectional associations of novel blood-based biomarkers of neurodegeneration, latent cognitive abilities and accelerometry-measured physical activity in healthy older adults. The analytical results revealed some noteworthy patterns concerning the role of plasma NfL in healthy older adults, which we believe could be useful for future hypothesis generation. Below, we discuss each pattern of results in turn.

When Aβ42/Aβ40 ratio, ptau-181, GFAP and NfL, were set to predict cognition, a significant association was detected between higher NfL concentrations and worse working memory. This is consistent with previous work showing negative associations between NfL and global cognition in non-demented older adults^49,50^, as well as negative associations with performance on single tests tapping different cognitive domains in healthy older adults, including memory and executive function^51^. Importantly, the analytical approach adopted here arguably increases the confidence assigned to such an association. First, the association with NfL was detected in a context in which the other three neurodegenerative markers were statistically controlled for, indicating a unique influence of NfL on working memory. Second, since working memory was modelled as a latent variable, from six individual tests of working memory updating, we can be more confident that the association with NfL is due to variability in task-general working memory ability and not to task-specific variability. However, it should also be noted that the association was not significant after adjustment for age, sex and education in the present study, which warrants some caution for its interpretation.

The apparent importance of NfL for normal cognition in aging, relative to the more AD-specific Aβ42/Aβ40 ratio, ptau-181 and GFAP, could be due to its more general role as a marker of neuro-axonal damage. Indeed, since NfL is primarily located in long myelinated axons^9^, higher NfL concentrations should most closely reflect white-matter changes^52^, which in turn is one of the most prominent pathologies in normal aging^53^. White matter is also central to age-related decline in executive function^54,55^, which incorporates the working memory updating construct in the present study^56^. The demonstrated association between NfL and working memory may therefore be reflective of the more advanced progression of white matter pathology, relative to AD-related pathology, in the cognitively normal study population, and its influence on working memory ability. As such, future research should continue to evaluate plasma-NfL as a potential marker of white matter deterioration, which could help shed light on its potentially domain-specific contribution to cognition in healthy aging.

The present study additionally probed the interactive associations of physical activity and plasma neurodegenerative markers with cognition in healthy aging. MVPA and NfL were found to interact in their association with episodic memory, also in the model with adjustment for demographic variables. When using NfL as moderator in the visualization of the effect, the interaction appeared to reflect a weakening of the favorable association between MVPA and episodic memory at higher levels of NfL. This particular interaction is consistent with a previous study reporting a dampening of the favorable association between MVPA and cognition at high NfL levels in 465 community-dwelling older adults^32^. It is also consistent with previous findings from the Chicago Health and Aging Project of a stronger association between moderate levels of physical activity and slower age-related cognitive decline at low NfL concentrations^36^. An attenuation of the positive association between physical activity and cognition at higher NfL concentrations suggests that physical activity may offer protection against the impact of neurodegeneration on old-age cognition, but only up until a certain threshold of neurodegeneration. Importantly, however, when MVPA was used as moderator in the visualization, an alternative or perhaps complementary interpretation emerged of the same effect. Specifically, the unfavorable association between NfL and episodic memory appeared to be strengthened at higher levels of MVPA, suggesting that a potentially negative impact of NfL on old-age cognition may be worsened at higher levels of physical activity. Taken together, the results call into question the proposal that physical activity benefits old-age cognition by acting directly to decrease neurodegenerative markers^26^. We speculate that the parallel neurotrophic effects of physical activity for promoting axonal regeneration in aging may be sufficient for counteracting subtle but not more pronounced white matter pathology in aging^57,58^, whilst also acknowledging the possibility that a higher level of physical activity may not always be unequivocally beneficial for cognition in geriatric populations^59^.

The present study additionally detected an association between higher NfL concentrations and *higher* levels of MVPA, contradicting previous findings as well as a direct effect of physical activity in reducing neurodegeneration^26,36^. Whilst this finding is unexpected and indicative of a negative effect of MVPA on NfL, the interaction between MVPA and NfL on episodic memory suggests that both factors need to be considered to understand their influence on cognitive ability. In this context, it should also be emphasized that the present study used accelerometers to measure habitual physical activity, which may have captured different aspects of movement behavior relative to the self-report measures in previous studies^36^. Furthermore, non-linear associations between NfL and physical activity may have contributed to the unexpected association between high NfL and high MVPA in the present study^60^, necessitating analytical approaches that requires larger sample sizes.

The present study has several important limitations. The used data was derived from an already completed trial, which was powered to detect different effects than the ones investigated here^40^. Furthermore, given the exploratory nature of the present study, no correction for multiple models and statistical tests was applied. As such, the heightened risk of false positive as well as false negative results need to be considered when interpreting the findings. The study was also limited to measures of cognitive level, providing no information on age-related cognitive decline. Another limitation was that the quantification of plasma neurodegenerative marker concentration was unsuccessful in a few cases, and that extreme outlier values were present for Aβ40. Finally, given the experimental aim of the original study, the diversity of the study population was restricted, which limits the generalizability of the results to a broader older population. However, the study also has some important strengths relative to previous research. Habitual physical activity was not reliant on self-report but was measured using a 7-day accelerometry assessment, which makes the study less susceptible to recall and social desirability biases^37^. The study also included several markers of neurodegeneration, which allows for a broader evaluation of their relative importance for physical activity and cognition. Finally, by using 14 individual tests to model four cognitive domains in latent space increases our confidence that the models actually captured the underlying cognitive abilities of interest, and not just test-specific performance^61^.

Taken together, the results of the present study indicate that NfL may be a particularly important plasma neurodegenerative marker for understanding normal cognition in aging, relative to more AD-specific markers of neurodegeneration. In regards to physical activity, the results suggest that the protective effect of physical activity for cognition in old age may be limited to lower levels of NfL and corresponding neuro-axonal damage, and that a higher level of physical activity may not always be optimal for reducing the cognitive consequences of NfL, which are both possibilities that should be considered in future confirmatory work.

## Supplementary Information

Supplementary Information.

## Data Availability

Swedish data protection laws prohibit us from putting the data in the public domain, but data and/or analyses can be made available from the corresponding author on reasonable request (Jonna.nilsson@gih.se). For requests that involve specific and well-defined analyses that are in line with the original ethics approval, a data use agreement can be used to effectively transfer the confidentiality obligations of the institution at which the original research was conducted to the institution of the recipient of the data.
