# Supplementary material for: Plasma markers of neurodegeneration, latent cognitive abilities and physical activity in healthy aging
Source: Sci Rep. 2024 Sep 17;14:21702. doi: 10.1038/s41598-024-72806-0 (PMC11408627; doi:10.1038/s41598-024-72806-0)
Supplement: Supplementary file 1 — Supplementary Information. [file 41598_2024_72806_MOESM1_ESM.docx]

**Supplementary materials for:**

Plasma markers of neurodegeneration, latent cognitive abilities and physical activity in healthy aging

Jonna Nilsson, Yiwen Jiang, Malin Johannesson, Marcus Moberg, Rui Wang, Susanne Fabre, Martin Lövdén, Örjan Ekblom & Maria Ekblom

**Supplementary materials 1: Pre-experiment**

Data from the pre-experiment testing stability of the proteins across freeze-thaw cycles. Points reflect individual data values. Dashed lines represent the three old participants and the solid lines represent the five young participants. Values with coefficient of variation above 20% and values below the lower level of quantification have been excluded.


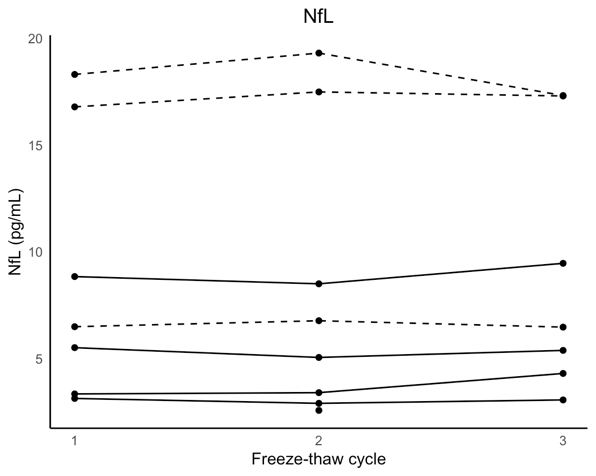

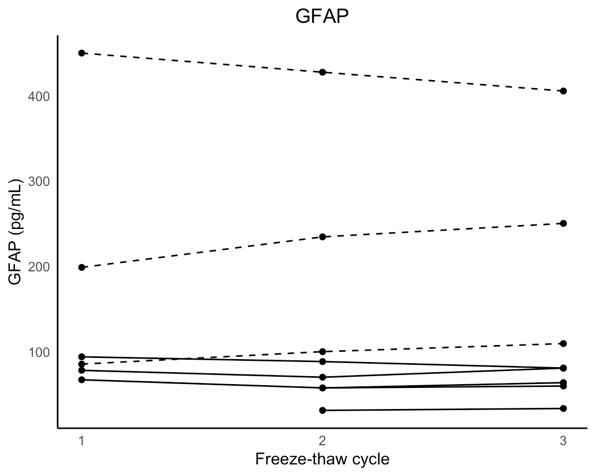


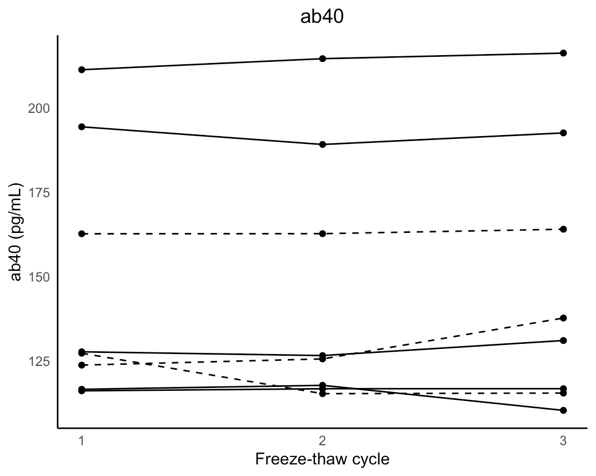

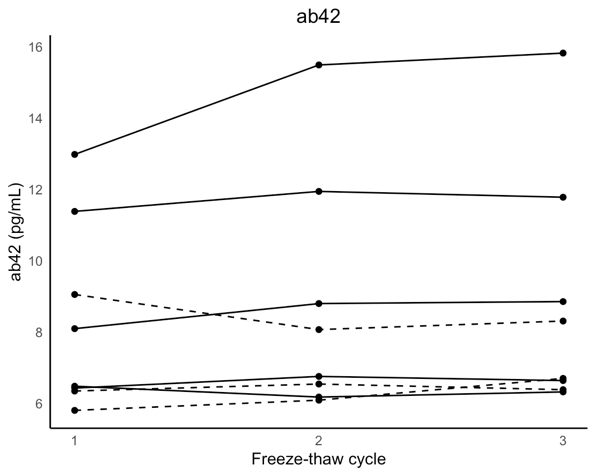


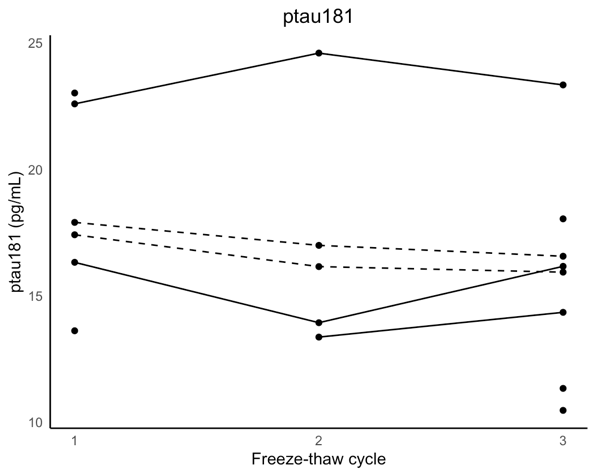


**Supplementary materials 2: Correlations**

Pearson’s correlation coefficients for associations between plasma neurodegenerative markers (pg/ml), age (years), BMI (kg/m^2^), MMT (Mini Mental State Exam score), MVPA (proportion daily moderate-to-vigorous intensity physical activity), LIPA (proportion daily light intensity physical activity), SED (proportion daily sedentary behavior). Sample sizes range from 73 to 93. * p<.05, ** p<.001.

**Supplementary materials 3: Extended sensitivity analysis with BMI as covariate**

Considering the inverse relationship between NfL and BMI, BMI was included as a covariate in an extended sensitivity analysis, additional to age, sex and education. For the additionally adjusted neurodegenerative marker prediction model, model fit was still satisfactory, χ2 (151, N=93)=178.64, CFI=0.95, RMSEA=0.044, and the path between NfL and working memory was just not significant, χ2 (1, N=93)= 3.77, p=0.052, and with a similarly sized standardized estimate (β=-0.23, 95% CI [-0.50, 0.003]). For the additionally adjusted physical activity interaction model, model fit was bordering on being unsatisfactory, χ2 (147, N=93)=181.85, CFI=0.94, RMSEA=0.05, and the interaction term between NfL and MVPA on episodic memory was still significant, χ2 (1, N=93)= 9.58, p=0.002, with a similarly sized standardized estimate, β=-0.38, 95% CI [-0.61, -0.14]. The covariance between MVPA and NfL was also significant, χ2 (1, N=93)= 3.99, p=0.46, with a positive path estimate (β=0.21, 95% CI [0.01, 0.41]).
